# CRISPR/Cas9: a tool to eradicate HIV-1

**DOI:** 10.1186/s12981-022-00483-y

**Published:** 2022-12-01

**Authors:** Ruchira Bhowmik, Binay Chaubey

**Affiliations:** Virology Lab, Centre for Advance Study, Department of Botany, University of Calcutta, 35 Ballygunge Circular Road, Kolkata, 700019 India

**Keywords:** CRISPR/Cas9, Gene editing, HIV-1/AIDS, Latent viral reservoirs, Antiretroviral therapy (ART)

## Abstract

The development of antiretroviral therapy (ART) has been effective in suppressing HIV replication. However, severe drug toxicities due to the therapy and its failure in targeting the integrated proviral genome have led to the introduction of a new paradigm of gene-based therapies. With its effective inhibition and high precision, clustered regularly interspaced short palindromic repeats (CRISPR)-associated protein-9 nuclease (Cas9) or CRISPR/Cas9 has emerged as an effective genome editing tool in the last decade. Mediated by guide RNAs (gRNAs), Cas9 endonuclease acts like genetic scissors that can modify specific target sites. With this concept, CRISPR/Cas9 has been used to target the integrated proviral HIV-1 genome both in in vitro as well as in vivo studies including non-human primates. The CRISPR has also been tested for targeting latent HIV-1 by modulating the proviral transcription with the help of a specialized Cas9 mutant. Overcoming the limitations of the current therapy, CRISPR has the potential to become the primary genome editing tool for eradicating HIV-1 infection. In this review, we summarize the recent advancements of CRISPR to target the proviral HIV-1 genome, the challenges and future prospects.

## Introduction

The human immunodeficiency virus/acquired immunodeficiency syndrome (HIV/AIDS) continues to be a major global health issue that has claimed ~ 36.3 million human lives worldwide [1]. According to the World Health Organization (WHO), by the end of 2020, globally ~ 38.0 million people were living with HIV with an estimate of ~ 1.5 million new infections [1]. With no vaccines on the shelf or in the pipeline, presently anti-retroviral therapy (ART) is the mainstay to reduce the viral load [2]. There are over 25 anti-viral drugs used in different combinations that have been effective in reducing the mortality and morbidity of HIV-1 infected individuals. However, these drugs do not target the integrated proviral genome in the host cell chromosome. Hence, the viral infection is not eradicated and the viremia rebounds once the therapy is stopped [3]. Therefore, the patients have to depend lifelong on the expensive therapy. Globally, so far only ~ 73% of infected individuals have access to proper antiretroviral therapy (ART) [1]. In the last few decades ART has made considerable improvement in the life expectancy of people infected with HIV. Present ART regimens have shown fewer side effects and effectively reduce viremia. However, they require life-long administration thereby causing several drug induced toxicities and comorbidities associated with aging [4, 5]. The general antiretroviral therapy comprises at least three antiretroviral drugs belonging to any of the four classes—nucleoside or nucleotide reverse transcriptase inhibitors (NRTIs or NtRTIs), non-nucleoside RT inhibitors (NNRTIs), protease inhibitors and fusion inhibitors [4]. Drug induced toxicity is one of the major causes of acute kidney injury found in people living with HIV [4]. Since, HIV itself increases the risk of chronic kidney disease (CKD), ART treatment is complicated in these individuals. The NtRTI Tenofovir disoproxil fumarate has shown to develop tubulopathy in 1–2% of recipients. The risk further increases with additional factors including diabetes, immunodeficiency, prolonged exposure or usage of ritonavir-boosted protease inhibitors [4]. Ritonavir also has cytotoxic effects leading to endoplasmic reticulum (ER) stress and mitochondrial dysfunction [5]. Additionally, liver diseases are one of the major co-morbidities related to ART as it accounts for 13% of deaths among the people living with HIV (PLWH). The older population is at a risk of mitochondrial dysfunction, non-alcoholic fatty liver disease and even liver cancer [6]. Cardiovascular disease (CVD) risk including heart failure and ischemic stroke still remains a major concern especially in patients treated with first generation ART [7]. Efavirenz and protease inhibitors have significant potential to develop into CVD and other associated metabolic disturbances [7].

These challenges get further accentuated due to the undetectable latent viral reservoirs consisting of inactive HIV proviral DNA in resting CD4 + T-cells that are established after infection [8]. Furthermore, the emergence of new viral mutants is inevitable due to the spontaneous mutations in successive replication cycles. Three polymerases contribute to viral replication–viral reverse transcriptase (RT), host RNA polymerase II (Pol II) and host DNA polymerase. Considering the high fidelity of the host DNA polymerase and its editing machinery, most of the errors are made by RNA Pol II and HIV-1 RT [9]. HIV-1 RT has a high error rate of 1 base per 3 × 10^5^ nucleotides incorporated (almost equal to that of RNA Pol II), resulting due to lack of proof-reading activity [10–12]. The subsequent diversity in HIV-1 mutants has enabled them to evade the host immune system and develop drug resistance [13]. Consequently, in addition to ART, several alternative therapeutic strategies have been explored to combat these challenges with varying success rates.

The ultimate cure for HIV-1 includes the permanent inhibition of viral replication without the requirement of lifelong administration of ART whereby individuals can lead a healthy life without the probability of recurring re-emergence of viremia. Among various approaches, one of the paradigms deals with effectively eradicating HIV-1 by targeting the integrated proviral DNA in infected cells. In recent years, this approach has been applied via genome editing. Initial studies targeted the HIV-1 LTR (long terminal repeat) by tailored recombinases based on the Cre-recombinase [14], which led to the excision of the proviral DNA from the cellular genome. Endonucleases like zinc finger nucleases (ZFN), transcription activator-like effector nucleases (TALEN) and homing endonucleases have been used to target specific DNA sequences of the proviral DNA [15, 16]. The nucleases induce double-stranded breaks (DSBs) that are repaired by the non-homologous end-joining (NHEJ) pathway frequently giving rise to indel mutations (insertions and deletions). However, in both ZFNs and TALENs, the rate of off-target effects and binding specificity are major challenges [17, 18]. CRISPR/Cas9, due to its high precision in inducing mutations, has evolved into a promising genome editing tool in the last decade [19].

The Cas9 endonuclease acts like a genetic scissor that introduces DSBs in the DNA at specific sites mediated by a guide RNA (gRNA) [20]. In its first application of CRISPR/Cas9 against HIV-1, the LTR region was targeted that successfully suppressed HIV-1 replication [21]. Efforts continued in this field with different approaches to target the proviral genome and a new mutant dCas9 to combat latent viral reservoirs. In this review, we summarise the various approaches and therapeutic applications of CRISPR/Cas9 in HIV-1/AIDS therapy and also highlight the limitations and future studies that are required in this field.

## CRISPR/Cas system in genome editing

### Brief overview

Discovered in 1987 [22], the CRISPR repetitive sequences were found to be derivatives of conjugated plasmids and bacteriophages. Using the “anti-sense RNAs as memory signatures” [19], CRISPR-Cas was able to introduce targeted DNA mutations in these pathogens leading to adaptive immunity in bacteria [23]. In 2012, Jinek et al. made breakthrough research where they introduced dual gRNAs to guide Cas9 endonuclease of *Streptococcus pyrogenes* for targeted DNA cleavage in vitro [24]. This discovery indicated that CRISPR-Cas9 could probably target any specific DNA in any organism.

**Fig. 1:**
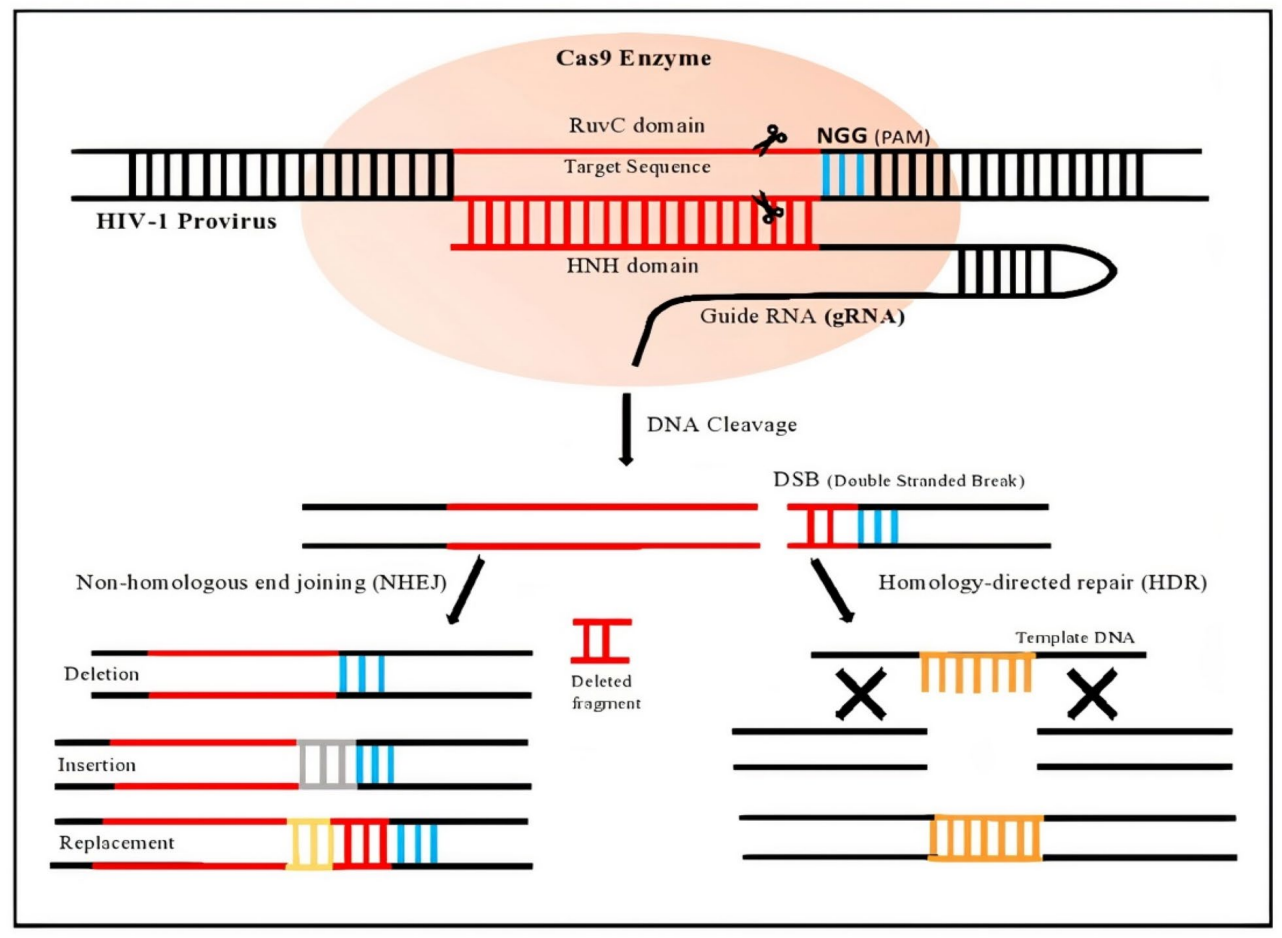
Schematic diagram of HIV-1 provirus gene editing by CRISPR/Cas9. Cas9 protein combined with sgRNAs introduces double-stranded breaks at specific regions. The breaks are repaired by two pathways; NHEJ which incorporates random indel mutations; and Homologous dependent repair (HDR) which introduces specific sequences with the help of donor templates

The specificity of CRISPR-Cas9 to a complementary sequence (PAM or *NGG* for spCas9) is mediated by 17–20 nucleotides present at the 5’ end of gRNA [20]. The sequence specificity provided by the gRNA-PAM prevents adverse off-target interactions. The two nuclease domains of Cas9, histidine-asparagine-histidine (HNH) and Recombination UV C (RuvC) cleave separate DNA strands (Fig. 1). The HNH domain cuts the target strand that is bound by the gRNA, while the RuvC domain cleaves the non-target strand [25]. Devoid of a template, this DSB is repaired via the NHEJ pathway by introducing random indel mutations [19]. Another alternative DNA repair pathway is the homology-directed repair (HDR) pathway which introduces well-defined mutations at a particular locus with an exogenous DNA repair template (Fig. 1) [19].

A double mutant of Cas9 enzyme involving the endonuclease domains results in a catalytically inactive or deactivated Cas9 (dCas9) that retains the gRNA-mediated DNA-binding specificity. This protein was shown to successfully fuse with transcription repressor or activator domains regulating the expression of target genes [26, 27]. Unlike other approaches (e.g., ZFNs and TALENs) that require substantial protein engineering of DNA-recognition domains for each DNA target site, CRISPR-Cas9 provides a relatively simple approach. With the advancement in delivery methods, multiple gRNAs have been used to target more than one target sequence improving the efficiency of gene targeting [28].

### Cas9 and other nuclease variants

The SpCas9 is a Type-II-A Cas9 protein that consists of two well-conserved nuclease domains, HNH and RuvC. Apart from SpCas9, several other nuclease variants have been isolated from different bacteria which can be used for gene editing processes providing insight into new delivery strategies, especially in in vivo studies.

SaCas9, derived from *Staphylococcus aureus* is ~ 1 kb shorter than SpCas9 and hence, suitable for packaging in AAV (Adeno-associated virus) vector for targeting HIV-1 provirus in in vivo systems [29]. Several CRISPR studies have used SaCas9-AAV systems for targeting LTR and other viral genes of HIV-1 in mice models and non-human primates (discussed later). Other nuclease variants include the Cas12a nuclease, formerly known as Cpf1 which can accommodate multiple crRNAs (crispr RNAs) under the transcriptional control of a single Pol III promoter [30]. While Cas9 produces blunt double-strand cuts, Cas12a produces staggered cuts in dsDNA. Additionally, the Cas13 nuclease which targets the RNA has been used in HIV-1 infected cells with significant results [31]. With the discovery of multiple nuclease variants, a varied usage of these Cas proteins can be employed in gene editing systems for getting effective results.

### Delivery methods

**Fig. 2:**
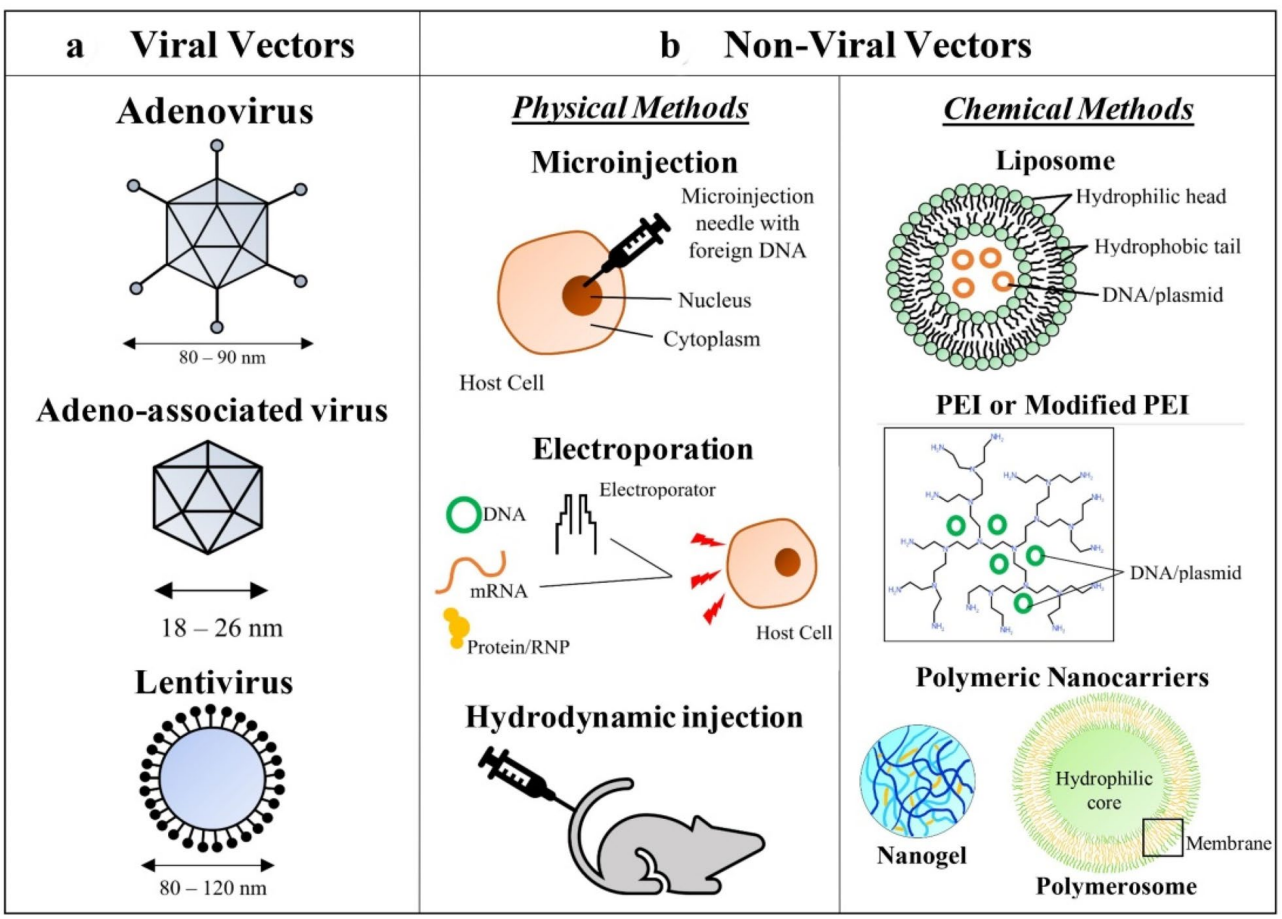
Vectors for CRISPR/Cas9 delivery. **A** Viral vectors include adenoviral, lentiviral and the adeno-associated viral (AAV) vectors. **B** Various physical and chemical methods can be used to deliver Cas9/sgRNAs. Microinjection and electroporation are mainly used in in vitro studies. The hydrodynamic tail vein injection is used for in vivo transfection of nucleic acids. Cas9/gRNAs can be delivered as RNPs via lipid-based nanoparticles like liposomes, polymeric nanocarriers and through PEI or modified PEI

There are several delivery options for the introduction of Cas9 and gRNA into the target cells. The components for delivery involve a DNA vector, gRNAs and Cas9 mRNA or Cas9/gRNA ribonucleoprotein (RNP) complexes [32–37]. The Cas9 and gRNAs can be delivered either as RNAs or can be encoded by a single construct in two separate plasmids. As shown in Fig. 2, the different delivery methods include electroporation, microinjection, cationic lipid and lipid-based nanoparticles [32, 36, 38, 39].

For in vivo applications, lipid nanoparticles or viral vectors like lentiviral vectors (LVs) or AAVs can be used [39]. However, the major concern with the viral vectors is the limited packaging capacity that restricts the efficiency of the delivery [40, 41]. In the case of LVs, smaller RNA sequences are seen to perform better and have improved transduction efficiency. Alternatively, the AAVs are smaller than LVs thus, making the packaging of the cassette even more challenging [42]. Although the use of smaller SaCas9 with AAV might be a solution, the efficiency of SaCas9 is less compared to SpCas9 [43]. In a recent study by Herskovitz et al. gRNAs designed against specific target sites delivered by lipid nanoparticles to the latently infected cells showed ~ 100% viral excision (discussed later) [44].

These delivery methods can establish either a non-integrative, transient expression or integrative, stable expression in the cells. While the stable expression of gRNA/Cas9 can prove advantageous in many experiments, in the long run, this may give rise to undesirable off-target activity [45]. Transient expressions lessen the safety concerns but won’t support long term CRISPR activity. A significant study by Liu et al. showed evidence of complete inactivation of proviral HIV-1 with repeated transfection of different Cas9 and Cas12a mRNA/protein sources with dual gRNAs in latently infected SupT1 T-cells [46]. Upon repeated Cas9 treatment, the viral rebound could no longer occur as the target sites were either mutated, excised or underwent inversion. However, there is ample scope for improving of the delivery of CRISPR/Cas in in vivo system.

## CRISPR/Cas mediated inhibition of HIV-1

### In-vitro studies

**Fig. 3:**
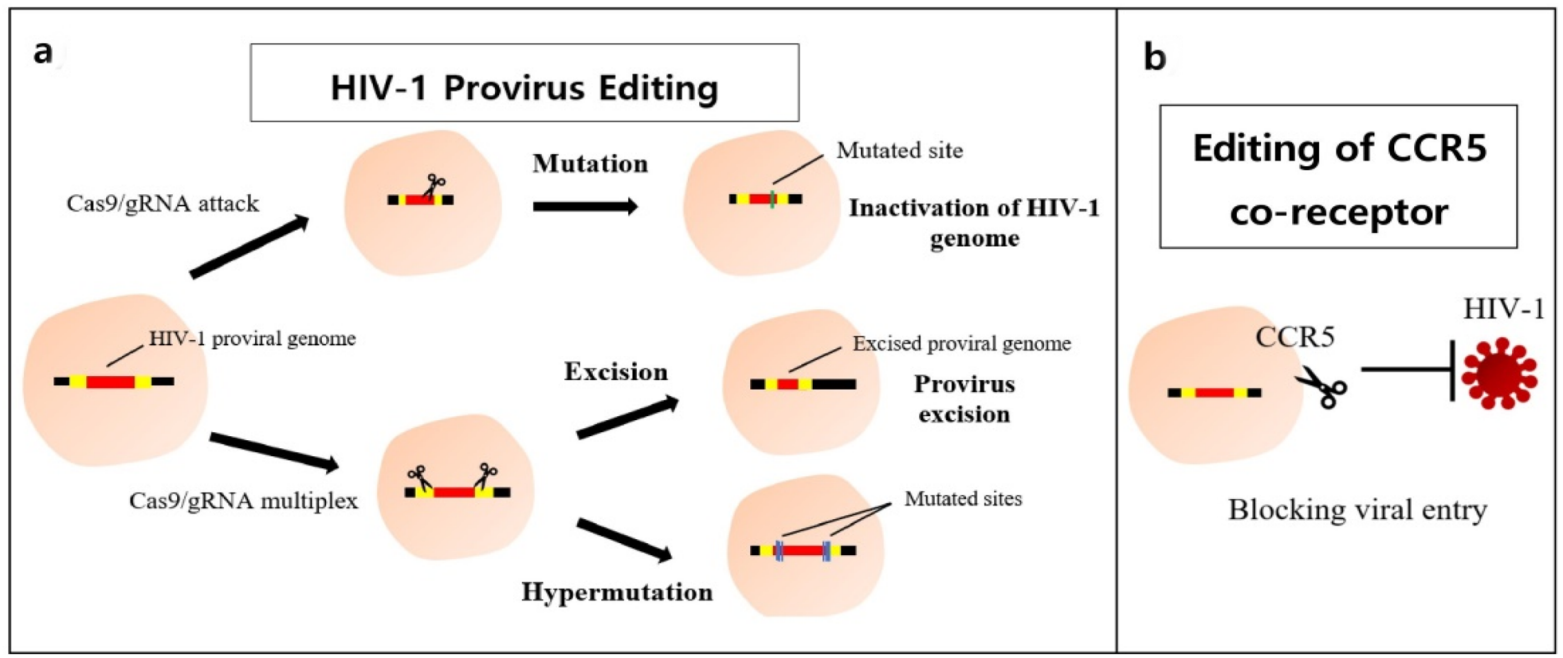
Gene editing by CRISPR/Cas9. **A** CRISPR/Cas9 introduces double stranded breaks in the HIV-1 LTR and/or viral genes thereby inactivating the proviral genome. Use of multiple gRNAs results in excision or hypermutation of the target sites. **B** The host co-receptors CXCR4 or CCR5 can also be targeted for blocking viral entry into the host cell thus, stopping further infection

CRISPR has emerged as an effective genome editing tool for targeting the HIV-1 genome in infected cells (Fig. 3). Ebina et al. provided the first proof of successful targeting of HIV-1 genome by CRISPR in infected HEK293T cells and HeLa cells. The study indicated the ability of CRISPR/Cas9 to effectively inhibit viral expression [21]. Liao et al. targeted multiple sites including the LTR regions and observed a decrease in protein expression regardless of the amount of integrated viral DNA [47]. In another study, Kaminski et al. placed the gene encoding Cas9 under the control of a Tat-activating promoter. The results showed cleavage of viral DNA indicating the Tat-mediated transactivation of the promoter for Cas9 expression [48].

In-vitro experiments were extended to other cell lines since the HIV-1 reservoir is not just composed of T-cells. One study showed that dual gRNAs complementary to the conserved regions of LTR excised a 9709 bp sequence in the latently infected promonocytes, microglial and T cells. There was absence of off-target activity and cytotoxicity; and the multiplex gRNAs in Cas9 transfected cells prevented HIV-1 infection [49].

To study the effect of CRISPR in latently infected cells, Zhu et al. used Jurkat cell lines latently infected by HIV-1. They designed gRNAs against 10 conserved sites and the tumour necrosis factor alpha (TNFα) was used to activate the viral gene expression [50]. The results showed a tenfold reduction in GFP reporter expression and ~ 20-fold reduction in p24 expression. Further, primary CD4 + T-cells isolated from healthy individuals were infected with HIV-1 and targeted at the LTR region by CRISPR-Cas9 [48]. A significant reduction in viral expression was observed in these cells. These experiments were extended to peripheral blood mononuclear cells (PBMC) and CD4 + T cells isolated from patients undergoing ART. In all the cases, there was an overall reduction of viral particles and expression of p24 and Gag proteins [48]. Specific gRNAs were designed against transcription factor binding sites (TFBSs) mainly NF-κB with a ‘high safety profile and broad-spectrum activity’. DSBs were observed via GUIDE-seq and no off-target activity was found in HeLa cells [51].

Lebbink et al. showed two sgRNAs for different target sites prevented viral replication and escape. This combinatorial approach was used to target viral matrix protein and three essential enzymes: reverse transcriptase, integrase and protease in latently infected Jurkat cells [52]. The dual-gRNA combinations were more effective in inhibiting viral replication. CRISPR-Cas9 treatment with dual gRNAs led to either of the three: hypermutation, excision or inversion [53]. Although excision could be detected, the viral inactivation mainly resulted from the acquisition of mutations in both the target sites inferring that hypermutation might be a major mechanism for HIV inactivation. Lentiviral vectors were also designed to contain three and six gRNAs that targeted the Tat and Rev regulatory elements of HIV-1 respectively. gRNA multiplexing against the viral Tat sequence in T cell line suppressed viral p24 protein and inhibited viral replication in the second round of infection and maintained protection for 45 days [54].

A significant obstacle to the HIV-1 cure is the viral diversity that results due to high rate of mutations [9]. This ultimately leads to immune evasion and resistance to antiretroviral drugs. Herskovitz et al. were able to develop a library of gRNAs capable of disrupting five unique HIV-1 exons, tat1-2, rev1-2, and gp41 [44]. The gRNAs were derived by identifying the consensus sequences targeting tat from sequence information of 4004 clinical strains of HIV-1. Multiple modes of delivery were used including transfection, electroporation, lentivirus and lipid nanoparticle (LNP). Results showed viral reduction in all the cases, 82% and 94% viral reduction was observed after transfection and lentivirus treatments, respectively. The multi-exon gRNA TatDE delivered by LNPs to the latently infected cells showed ~ 100% viral excision [44].

In addition to targeting the proviral DNA that is integrated into the host genome, few studies attempted to target the pre-integrated HIV-1 DNA in cytoplasm. In vitro studies by Liao et al. targeted the HIV-1 cDNA, synthesized by reverse transcriptase, to prevent its integration and further infection [47]. By using a GFP reporter gene, a reduction in positive cells was observed. Another study was performed on HIV-1 positive 293 T cells which were transduced with Cas9-NLS and gRNAs targeting the R and U5 regions of LTR [55]. A significant reduction of integrated and pre-integrated viral DNA was noted. However, no change was observed in the viral cDNA (early DNA) that was present in the cytoplasm [55]. Both the studies indicated that CRISPR/Cas9 can not only be used for the inactivation of proviral DNA but also the pre-integrated viral DNA to prevent its integration into the host genome, an observation with tremendous prophylactic potential.

A combination of SaCas9/gRNAs disrupted the HIV-1 genome more efficiently than a single sgRNA/SaCas9 [56]. Cas12a was assessed by Gao et al. due to its smaller size and better ability to accommodate multiple crRNAs under a single Pol III promoter [30]. Experiments with Cas12a showed more sustained antiviral activity in comparison to Cas9 [57]. The RNA-editing Cas13 system has been recently tested against HIV-1 infected cells. The Cas13d system was able to effectively inhibit HIV-1 infection in primary CD4 + T cells and also, suppressed reactivated HIV-1 from latently infected cells [31]. The CRISPR-Cas13a targets HIV-1 RNA leading to a reduction in viral gene expression. It not only inhibits the newly synthesized viral RNA from the proviral DNA but also targets the viral RNA that enters the host cells [58]. The CRISPR-Cas13 system provides an alternative approach for the treatment of HIV-1.

The CRISPR based studies prove that it not only inactivated the integrated HIV but also the pre-integrated viral DNA. A diverse range of delivery methods and nuclease variants have been used to show the significant potential that CRISPR possess in targeting the HIV-1 genome. A summary of the *in-vitro* studies has been made in Table 1.

**Table 1:** CRISPR/Cas Systems for targeting proviral HIV-1 (*In-vitro* studies)

CRISPR/Cas System	Cell type	Target region	Delivery	Results	References
SpCas9	HeLa, HEK293T, Jurkat	T5 site of TAR seq in the R region T6 site of NF-kB seq of the U3 region	Transfection	45.6% to 20% decrease in proviral gene expression in 293 T cells receiving T5 gRNA. Target site showed indel mutations	Ebina et al. [21]
SpCas9	HEK293T	LTR (R region)	Lentivirus	Decrease in protein expression regardless of the amount of integrated viral DNA	Liao et al. [47]
SpCas9	TZM.B1	LTR	Transfection	Cleavage of viral DNA	Kaminski et al. [48]
SpCas9	CD4 + T cells PBMC (From patients)	LTR	Transfection	Decrease in viral cDNA number; Reduction in viral particles and expression of p24 and Gag proteins	Kaminski et al. [48]
SpCas9	CHME5, TZM-Bl, U937	LTR	Transfection	gRNA-Cas9 complex excised a 9709 bp sequence between 5’ and 3’ LTR sequences	Hu et al. [49]
SpCas9	Jurkat (JLat 10.6)	LTR region, *pol*, *rev* (2nd exon)	Transfection	tenfold GFP reduction and 20-fold p24 reduction according to the respective gRNAs	Zhu et al. [50]
SpCas9	HeLa, Jurkat, TZM-bl	LTR (NF-κB Binding Sites)	Transfection Lentivirus	DSBs observed via GUIDE-seq, absence of off-target activity, reduction of 5’ LTR-driven HIV-1 transcription	Chung et al. [51]
SpCas9	Jurkat	LTR and viral genes	Lentivirus	Reduction of viral replication with dual gRNAs	Lebbink et al. [52]
SpCas9	HEK293T, SupT1 T cells	*Gag, tat/rev, env*	Lentivirus	Dual gRNAs treatment led to either of the three: hypermutation, excision or inversion	Binda et al. [53]
SpCas9	MT-4 T cells HEK293T	*Tat/Rev*	Lentivirus	gRNA multiplexing against Tat in T cell line suppressed viral p24 protein and inhibited viral replication	Ophinni et al. [54]
SpCas9	293 T cells	LTR (R and U5)	Transfection	Three to five-fold reduction in integrated viral DNA, two-fold in late DNA and no change in early DNA	Yin et al. [55]
SpCas9	CD4 + T Cells Monocytes HEK 293FT Jurkat, ACH2 T cells	Exons *(tat1-2, rev1-2,* and *gp41*)	Transfection Electroporation Lentivirus Lipid nanoparticle (LNP)	Multi-exon gRNA TatDE delivered by LNPs showed 100% viral excision	Herskovitz et al. [44]
SaCas9	Jurkat C11 cells TZM-bI	LTR and viral genes	Lentivirus	Combination of SaCas9/gRNAs disrupted the HIV-1 genome more efficiently than a single sgRNA/SaCas9. Dual or Triple gRNAs in an all-in-one lentiviral vector reduced viral production	Wang Q et al. [56]
Cas12a	HEK293T, SupT1 T cells	LTR	Lentivirus	Cas12a shows superior antiviral activity, achieve full HIV inactivation with only a single gRNA	Gao et al. [30]
SpCas9 Cas12a (Transient)	SupT1 T cells	*Gag, tat/rev*	Lentivirus	Complete inactivation of proviral HIV-1 with repeated transfection of different Cas9 and Cas12a mRNA/protein sources with dual gRNAs	Liu et al. [46]
Cas13d	CD4 + T cells	*Gag, pol,* protease, integrase	Lentivirus	Effectively inhibited HIV-1 infection and also, suppressed reactivated HIV-1 from latently infected cells	Nguyen et al. [31]
Cas13a	HEK293T, JLat 10.6	HIV-1 RNA	Lentivirus	Reduction in viral gene expression. Not only inhibits the newly synthesized viral RNA from the proviral DNA but also targets the viral RNA that enters host cells	Yin L et al. [58]

### Animal models

In vitro studies have shown the advantages of CRISPR/Cas9 in targeting the proviral DNA in latently infected cells. However, in vivo application of this approach remains challenging. One of the early in vivo studies was performed by Kaminski et al. on transgenic Tg26 mice which harboured integrated HIV genome in different tissues [29]. They used AAV9 vectors to deliver the SaCas9 and gRNAs that targeted the LTR and *gag* regions. HIV-1 Tg26 mice were injected twice by the tail vein at an interval of five days and later the DNA isolated from various tissues was studied. The study demonstrated the excision of target sequence and 80–90% reduction of *gag* and *env* RNA, respectively, in circulating lymphocytes thus, providing the first evidence of HIV-1 obliteration in in vivo studies [29].

Successive studies by Yin et al. demonstrated increased inhibition of proviral transcription and replication in different mice models by using multiplex gRNAs [59]. An all-in-one AAV with a combination of SaCas9 and quadruplex gRNAs targeting LTRs and other genes were injected into Tg26 mice. Deletions at target sequences in HIV-1 genome were detected in samples from spleen, liver and bone marrow. Excision and reduction of HIV gene expression were also found by intravenous injection of SaCas9/sgRNAs AAV-DJ/8 in Tg26 mice [59]. An NCr nude mouse was infected with the AAV-SaCas9 vector and EcoHIV-eLuc (a chimeric HIV-1 virus that switches the gp120 gene with a gp80 gene from mouse leukemia virus). Further, results of provirus excision were detected in the brain, colon, heart, spleen and lung in the clinically relevant BLT mice. The humanized bone marrow/liver/thymus (BLT) mice were intravenously injected with the HIV-eLuc reporter virus followed by the delivery of AAV-SaCas9 vector [59]. Like previous mice models, excision of target sequences was observed in the proviral DNA in different tissues. This study of CRISPR treatment in three different mice models demonstrated strong potential of CRISPR treatment in future clinical studies [59].

A recombinant AAV with dual gRNAs targeting the *gag* and LTR regions with SaCas9 showed cleavage and excision of integrated provirus in transgenic mice [29]. Bella et al. isolated PBMCs from HIV-1 positive individuals undergoing ART and injected them into NRG rats [60]. Multiplex gRNAs targeting LTR regions were delivered with Cas9 to the animals using a lentiviral vector and ~ 90% reduction of viral DNA and excision of the fragment between the target sequences were observed [60].

Further studies were performed by Mancuso et al. in non-human primates (rhesus macaques) that were infected with Simian immunodeficiency virus (SIV) [61]. Delivery of AAV9-SaCas9 vectors designed for targeting the SIV genome showed a significant reduction of viral DNA in tissues. The target sequences showed precise cleavage and excision in samples collected from infected blood cells and other tissues including lymph nodes, spleen, bone marrow and brain [61].

In a combinatorial study with ART and CRISPR-Cas9 system, Dash et al. demonstrated elimination of HIV-1 in mice models [62]. A humanized mouse was first infected with HIV-1 that was treated with long-acting slow-effective release (LASER) ART which had enhanced lipophilicity to penetrate the viral reservoirs followed by a slow-release thereby decreasing the frequency of administration. An AAV9-SaCas9 vector with dual gRNAs targeting LTR and gag region was injected intravenously into the infected mice. The analysis of viral DNA and RNA after treatment showed effective decrease in viral load in dual gRNA treated mice compared to mice singularly treated with LASER ART or CRISPR alone [62].

In vivo studies have shown usage of several mice models to target the integrated proviral genome. Multiplex gRNAs in these models have shown significant viral reduction and excision of proviral DNA. Research on non-human primates infected with SIV has shown viral reduction and excisions in tissues collected from various organs. Additionally, a combinatorial therapy of CRISPR and ART in a humanized mouse showed decrease in viral load. Although some of these in vivo studies have shown encouraging result, the viral rebound has also been observed in some. More research is required to overcome the challenges of viral escape and rebound in in vivo studies. A summary of in-vivo studies has been presented in Table 2.

**Table 2:** CRISPR/Cas systems for targeting proviral HIV-1 (*In-vivo* studies)

CRISPR/Cas system	Organism	Target region	Delivery	Results	References
SaCas9	Tg26 mice	LTR and viral genes	AAV9 (Adeno-associated vector)	Deletion at the target sequences in all the tissue samples studied. Excision of target sequence, reduction of 80–90% *gag* and *env* RNA	Kaminski et al. [48]
SaCas9	Tg26 mice NCr nude mouse BLT mice	LTR and viral genes	AAV (Adeno-associated vector)	Deletions at target sequences in samples collected from spleen, liver and bone-marrow. Excision and reduction of HIV gene expression	Yin et al. [59]
SpCas9	NRG rats	LTR and *gag*	Lentivirus	Cleavage and excision of integrated provirus in between the target sites	Bella et al. [60]
SaCas9	Humanized Mice (Engrafted with human CD34 + HSC)	LTR and *gag*	LASER ART therapy + AAV9 (Adeno-associated vector)	Effective decrease in viral load in dual treated mice compared to mice that were singularly treated with LASER ART or CRISPR alone In two of the seven mice, the viral load was undetectable	Dash et al. [62]
SaCas9	Rhesus macaques	LTR and *gag*	AAV9 (Adeno-associated vector)	Significant reduction of viral DNA in the blood and tissues. Precise cleavage and excision in samples collected from infected lymph nodes, spleen, bone-marrow and brain	Mancuso et al. [61]

## Use of dCas9 as a modulator of provirus transcription

The major obstacle while tackling HIV-1 infection is the inability to detect and target the latently infected cells. Latency is easily established in activated CD4 + T cells that are *‘reverting to a resting memory state’* or EMT cells (effector-to-memory transitioning cells) due to the presence of dNTPs for reverse transcription, high CCR5 expression and sequestration of activation-dependent host transcription factors (i.e., NF-κB and NFAT) [63]. These infected cells are unable to undergo lysis and produce virions as the expression of the integrated proviral genome is transient and minimal. Hence, these cells can easily evade immune effector mechanisms and enter a state of latency [63]. Due to the absence of a suitable marker to detect the latently infected cells, targeting them remains a major challenge in establishing a permanent cure for HIV infection.

**Fig. 4:**
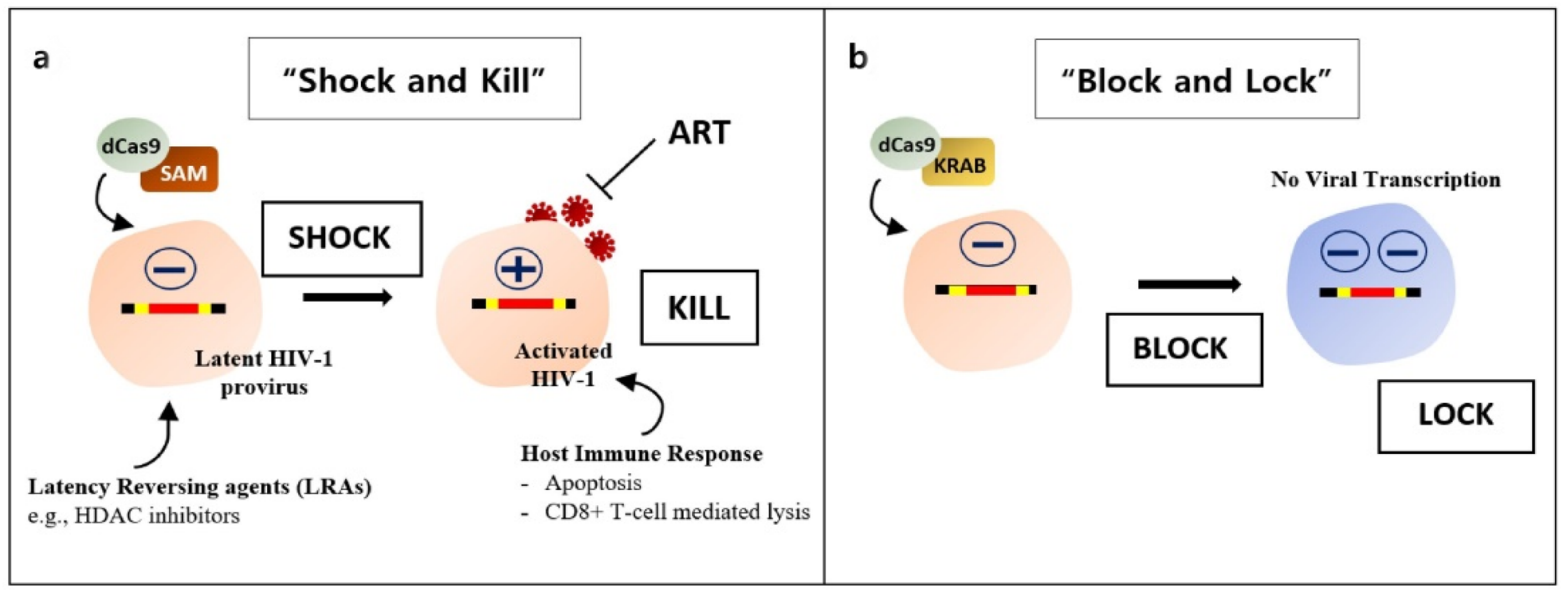
Modulation of proviral gene transcription by combining dCas9 either with transcriptional activators or repressors. **A** “Shock and kill” approach: dCas9 combined with transcriptional activator SAM is used for activating the latently infected cells. Once activated, the host immune response “kills” the cells. **B** “Block and lock” approach: dCas9 combined with transcriptional repressor KRAB is responsible for permanently inactivating the proviral gene transcription

The CRISPR based system has the potential to edit the proviral gene both in in vitro as well as in vivo systems. Hence, several authors took to CRISPR for approaching the undetectable HIV-1 latency. One of the strategies is the “shock and kill” approach (Fig. 4) which is employed to reactivate the latent viral reservoirs and purge them either through the host’s immune response or the presence of ARTs [64]. The latent reservoirs are larger than originally anticipated and driven by stochastic events in both active and resting memory T cells. Hence, the cell reactivation agents or HDAC inhibitors alone are unlikely to reactivate the latency [65]. A combination of latency-reversing agents (LRAs) and ART have better efficiency in eliminating the HIV-1 latent reservoirs [65]. However, not all viral reservoirs are eradicated and the toxic off-target effects of these drugs have led to additional strategies for reactivation of latent reservoirs [66].

Transcriptional activation using various forms of engineered CRISPR/Cas9 was directed to an activation ‘hotspot’, located within 200 bp upstream of the HIV transcriptional start site (TSS). For activation of this region, dCas9 fused with VP64 (C-terminal transcription activation domain of herpes virus) was used which along with multiple sgRNAs boosted the expression of target genes [27]. In HIV-1 reactivation studies, dCas9 was fused with a transcriptional activator domain that could activate the latent viral reservoirs [67]. Further studies using a synergistic activation mediator (SAM) system that recruited multiple transcriptional activation domains to a DNA target using specific gRNAs and dCas9 induced robust transcriptional activation of HIV-1 genomes. Results were analysed not only at RNA and protein level but also by the release of infectious virion particles [68]. A similar result of activation of viral gene expression with dCas9-SAM was demonstrated by Limsirichai et al. [69]. The HIV-1 LTR promoter was activated when targeted with 7 sgRNAs designed against functional elements of viral LTR including the U3 region, NF- κB and Sp1 binding sites, R domain and U5 region [69]. While all 7 gRNAs induced gene activation, only 2 sgRNAs that targeted the NF- κB binding sites and the TAR elements stimulated expression of the latent genes of HIV-1. It was further demonstrated that the combination of dCas9-SAM with latency-reversal compounds can increase proviral activation in different cell lines [69]. Zhang et al. targeted the 3’ LTR region of HIV-1 by designing 20 sgRNAs. Two target sites present near or at the NF- kB binding sites showed high efficiency and specificity in inducing reactivation of latent viral reservoirs in Jurkat cells, CJME5 microglial cells and TZM-B1 epithelial cells [67]. Saayman et al. also found strong activation sites near NF- kB binding sites while targeting the 3’ LTR region. This activation system showed better response and efficiency than the latency-reversing compounds in latent T cell lines [70].

Meanwhile, the “block and lock” approach takes a more permanent stance by blocking the viral rebound (Fig. 4) [71]. One of the studies employing this approach used dCas9 fused with a repressor domain Kruppel-associated box (KRAB) which actively repressed gene transcription. By inducing cell stimulation by LRAs, the dCas9-KRAB expressing lymphoblastoid T cells showed ~ 60% reduction in HIV-1 expression [72]. Liao et al. showed that dCas9-KRAB was able to repress the expression of the provirus when the gRNAs targeted the Repeat (R) domain but not with U3 or U5 sequences [47].

To summarize, both the “shock and kill” and “block and lock” approaches using CRISPR have significant potential as treatment for HIV-1. The “shock and kill” strategy using dCas9 is sequence-specific with a lesser off-target activity which is a noteworthy improvement over the current drug therapies. However, more investigation is needed to understand the eradication of proviral genome upon reactivation of viral reservoir. Since HIV-1 can evade host immune responses and antiretroviral therapies, viral rebound is an inevitable phenomenon. Lastly, since most of these studies have been conducted in vitro, further in vivo experiments need to be conducted to make it a therapeutic reality.

## Limitations of the CRISPR/Cas System

Although CRISPR/Cas9 has shown promising results in inhibiting and even excising the proviral HIV-1, several issues need to be addressed. As discussed earlier, a major challenge while tackling HIV-1 is its high mutation rate leading to a variety of strains [9]. These mutations in the target sequences may interfere with the Cas9 cleavage efficiency. Single nucleotide mismatches with the gRNA in PAM proximal region of the target DNA may reduce Cas9 cleavage activity [73]. Therefore, highly conserved regions among different HIV-1 strains can be targeted. The study of Herskovitz et al. provides an insight into this challenge as the gRNAs designed for the experiment were derived by analysing the consensus sequences of 4004 clinical strains of HIV-1 [44]. However, escape variants are also produced after Cas9 cleavage activity and its subsequent repair [74]. Studies by Wang G et al. and Wang Z et al. explained the mechanism of HIV-1 escape from CRISPR/Cas9 treatments [75, 76]. The experiments were performed in CD4 + T cells treated with Cas9 and gRNAs targeting specific regions of the proviral genome. Though the experiment showed an overall virus inhibition, viral rebound was observed in all these cases. The cells treated with gRNAs targeting lesser conserved regions showed high levels of HIV-1 production after a certain time while it took longer if treated with gRNAs against conserved sequences. Sequencing of the target regions indicated mutations at the recognition site of gRNA [75, 76].

Later studies have shown that the resistant mutations mainly appeared at the Cas9 cleavage site where the DNA repair takes place [77]. This suggested the involvement of the repair mechanism NHEJ in inducing these mutations during the repair process.

Another obstacle with CRISPR is off-target activity. CRISPR can tolerate imperfections in the RNA–DNA duplex thereby giving rise to unintended off-target activity [78]. Reduction of off-target effects is crucial as it may induce mutations in essential genes, tumour-suppressor genes or chromosome translocations, leading to severe consequences [79]. Though the off-target cleavage by Cas9 is limited as compared to other nucleases [80], significant off-target phenomenon was reported for gRNAs containing 6 or more mismatches [81]. Several strategies have been employed to counter this problem including the creation of bioinformatic tools to design gRNAs and predict their off-target activity. Efforts were made to reduce this by using truncated gRNAs [82], paired Cas9 nickase [83] and dimerization-dependent RNA-guided FokI-dCas9 nucleases (RFNs) [84]. Degradation of the Cas9/gRNA complex after the genome editing will leave no non-specific footprints. However, this approach triggered innate immune responses leading to cytotoxicity. Hence, an in-depth assessment of Cas9 immunogenicity is required for further understanding of this issue [3].

Delivery of large CRISPR/Cas9 complex poses another challenge. The viral vector-based delivery systems include lentiviral, AAV and adenoviral vectors. Due to its capacity to incorporate large DNA fragments, adenoviral vectors have been used in many CRISPR/Cas9 applications [42]. Lentiviral vectors have high efficiency as a delivery tool but their random integration into the genome is a concern [38]. AAVs have low toxicity and are relatively safe, but their small packaging size reduces expression and efficacy [37]. Other delivery systems including polymer polyethyleneimine (PEI), lipid-based reagents and nano-particles can be potential options [33, 35, 38].

## Conclusion

A major revolution in the field of genome editing occurred with the introduction of CRISPR/Cas9 which can effectively manipulate genes in cell culture systems to newly engineered transgenic animal models. With its high precision, expedient design and low off-target activity, CRISPR/Cas9 possesses the potential for eradication of HIV-1. ART, which continues to encounter drug resistance, side effects, high cost and lifelong administration, has remained our only defense against HIV infection so far. Although the success of ART exceeds our expectations with the reduction of viral load to almost undetectable levels, its inability to permanently remove the provirus affects the aging HIV-infected population leading to HIV-related complications as well as drug-induced toxicities [5]. CRISPR/Cas9 provides a new paradigm for solving some of the fundamental barriers posed by HIV infection.

In this review, we summarized some of the significant research in the field of genome editing of HIV-1 by CRISPR/Cas9 in different cell lines and animal models. With the help of appropriate delivery vectors, it can specifically target multiple genes with a relatively simple design of gRNAs. CRISPR/Cas9 was reported to successfully induce mutations or excisions in the proviral genome in latently infected cells. In studies held in patient-derived cells [48] and non-human primates [61], > 90% reduction in viral copy number was achieved. Herskovitz et al. achieved ~ 100% viral excision while working with multi-exon gRNAs which were delivered to latently infected cells by lipid nanoparticles [44]. In addition to the excision and deactivation of the proviral genome, CRISPR/Cas9 was able to target the non-integrated genome too [47, 55].

A major challenge to this approach is the emergence of viral escape mutants. Recent studies have shown that the ‘indels’ which should be able to inactivate the virus might aid in the escape mechanism. The virus might continue to replicate and infect the neighbouring cells. This change in the virus will be undetectable by the same machinery and becomes resistant to any future attacks [75, 76]. Multiplex gRNAs or application of combinatorial therapy of drugs and CRISPR can address this issue.

Another area of concern is the delivery system of Cas9-gRNAs. Electroporation and microinjection have shown positive results in in vitro systems, however, applying them to in vivo models are not suitable. LVs and AAVs have been used both in in vitro as well as in vivo systems. However, the packaging efficiency of these viral vectors remains a challenge. The large size of SpCas9 is a challenge for effective delivery. Recent studies have shown the use of alternate forms of nuclease like SaCas9, Cas12a and even the RNA editing Cas13a providing significant results. However, further study is needed to evaluate its efficiency in proviral genome editing. Alternate forms of delivery like lipid-nanoparticles are effective in treating the latently infected cells. Additionally, a combinatorial therapy using ART along with an effective delivery system of CRISPR has been shown to reduce the viral load in different mice models [62]. Even though further studies are required, the above observations give a strong foundation to address the challenges posed by the application of CRISPR in HIV-1 infection.

Additionally, the mutant dCas9 is a promising approach for reactivation of latent viral reservoirs with specific target activation. Several dCas9 systems have shown great potency in reactivating the latent viral reservoirs without any off-target effect, unlike the LRA drugs. These approaches are still in their initial stages of in vivo studies, they certainly show the potential for eradication of viral infection.

With ~ 38 million people living with HIV-1, CRISPR/Cas9 brings a new hope to eradicate the infection. While the search for new developments continues, several issues need further investigation for future applications: (1) Reduction of off-target activity; (2) Understanding the mechanism of viral escape from genome editing; (3) Identification and characterization of cells that contain the latent HIV- provirus and (4) Effective delivery system. Considering the potential of the CRISPR/Cas9 approach and the persisting questions, the research and developments for its therapeutic application for the eradication of HIV-1 have a promising future.

## Data Availability

This is a review article only and no materials used except the research articles.

## Abbreviations

AAV: Adeno-associated virus
AIDS: Acquired immunodeficiency syndrome
ART: Antiretroviral Therapy
CRISPR: Clustered regularly interspaced short palindromic repeats
crRNAs: Crispr RNAs
DSB: Double-stranded breaks
HDAC: Histone deacetylases
HDR: Homology-directed repair
HIV: Human immunodeficiency virus
KRAB: Kruppel-associated box
LRA: Latency reversing agents
LTR: Long terminal repeat
LV: Lentiviral vectors
NHEJ: Non-homologous end-joining
NNRTIs: Non-nucleoside reverse transcriptase inhibitors
NRTIs: Nucleoside reverse transcriptase inhibitors
NtRTIs: Nucleotide reverse transcriptase inhibitors
PAM: Protospacer adjacent motif
PBMC: Peripheral blood mononuclear cells
PEI: Polyethyleneimine
PLWH: People living with HIV
RNP: Ribonucleoprotein complexes
SAM: Synergistic activation mediator
SIV: Simian immunodeficiency virus
TALEN: Transcription activator-like effector nucleases
TFBS: Transcription factor binding sites
ZFN: Zinc finger nucleases
WHO: World Health Organization

## References

[1] Global HIV and AIDS statistics—fact sheet. UNAIDS. https://www.unaids.org/en/resources/fact-sheet. Accessed 13 Aug 2021.

[2] Palella FJ, Delaney KM, Moorman AC, Loveless MO, Fuhrer J, Satten GA (1998). Declining morbidity and mortality among patients with advanced human immunodeficiency virus infection. N Engl J Med.

[3] Xiao Q, Guo D, Chen S (2019). Application of CRISPR/Cas9-based gene editing in HIV-1/AIDS therapy. Front Cell Infect Microbiol.

[4] Wearne N, Davidson B, Blockman M, Swart A, Jones ES (2020). HIV, drugs and the kidney. Drugs Context..

[5] Ganta KK, Chaubey B (2019). Endoplasmic reticulum stress leads to mitochondria-mediated apoptosis in cells treated with anti-HIV protease inhibitor ritonavir. Cell Biol Toxicol.

[6] Chawla A, Wang C, Patton C (2018). A review of long-term toxicity of antiretroviral treatment regimens and implications for an aging population. Infect Dis Ther.

[7] Vos AG, Venter WDF (2021). Cardiovascular toxicity of contemporary antiretroviral therapy. Curr Opin HIV AIDS.

[8] Siliciano RF, Greene WC (2011). HIV latency. Cold Spring Harb Perspect Med.

[9] Abram ME, Ferris AL, Das K, Quinoñes O, Shao W, Tuske S, Alvord WG, Arnold E, Hughes SH (2014). Mutations in HIV-1 reverse transcriptase affect the errors made in a single cycle of viral replication. J Virol.

[10] Sanjuán R, Nebot MR, Chirico N, Mansky LM, Belshaw R (2010). Viral mutation rates. J Virol.

[11] Ji JP, Loeb LA (1992). Fidelity of HIV-1 reverse transcriptase copying RNA in vitro. Biochemistry.

[12] Roberts JD, Bebenek K, Kunkel TA (1988). The accuracy of reverse transcriptase from HIV-1. Science.

[13] Fraser C, Lythgoe K, Leventhal GE, Shirreff G, Hollingsworth TD, Alizon S, Bonhoeffer S (2014). Virulence and pathogenesis of HIV-1 infection: an evolutionary perspective. Science.

[14] Sarkar I, Hauber I, Hauber J, Buchholz F (2007). HIV-1 proviral DNA excision using an evolved recombinase. Science.

[15] Manjunath N, Yi G, Dang Y, Shankar P (2013). Newer gene editing technologies toward HIV gene therapy. Viruses.

[16] Stone D, Kiem HP, Jerome KR (2013). Targeted gene disruption to cure HIV. Curr Opin HIV AIDS.

[17] Sander JD, Dahlborg EJ, Goodwin MJ, Cade L, Zhang F, Cifuentes D, Curtin SJ (2011). Selection-free zinc-finger-nuclease engineering by context-dependent assembly (CoDA). Nat Methods.

[18] Juillerat A, Dubois G, Valton J, Thomas S, Stella S, Maréchal A (2014). Comprehensive analysis of the specificity of transcription activator-like effector nucleases. Nucleic Acids Res.

[19] Doudna JA, Charpentier E (2014). The new frontier of genome engineering with CRISPR-Cas9. Science..

[20] Cho S, Kim S, Kim J (2013). Targeted genome engineering in human cells with the Cas9 RNA-guided endonuclease. Nat Biotechnol.

[21] Ebina H, Misawa N, Kanemura Y, Koyanagi Y (2013). Harnessing the CRISPR/Cas9 system to disrupt latent HIV-1 provirus. Sci Rep.

[22] Ishino Y, Shinagawa H, Makino K, Amemura M, Nakata A (1987). Nucleotide sequence of the iap gene, responsible for alkaline phosphatase isozyme conversion in *Escherichia*
*coli*, and identification of the gene product. J Bacteriol.

[23] Mojica FJ, Díez-Villaseñor C, García-Martínez J, Soria E (2005). Intervening sequences of regularly spaced prokaryotic repeats derive from foreign genetic elements. J Mol Evol.

[24] Jinek M, Chylinski K, Fonfara I, Hauer M, Doudna JA, Charpentier E (2012). A programmable dual-RNA-guided DNA endonuclease in adaptive bacterial immunity. Science.

[25] Jinek M, Jiang F, Taylor DW, Sternberg SH, Kaya E, Ma E (2014). Structures of Cas9 endonucleases reveal RNA-mediated conformational activation. Science.

[26] Qi LS, Larson MH, Gilbert LA, Doudna JA, Weissman JS, Arkin AP (2013). Repurposing CRISPR as an RNA-Guided platform for sequence-specific control of gene expression. Cell.

[27] Gilbert LA, Larson MH, Morsut L, Liu Z, Brar GA, Torres SE (2013). XCRISPR-mediated modular RNA-guided regulation of transcription in eukaryotes. Cell.

[28] Kabadi AM, Ousterout DG, Hilton IB, Gersbach CA (2014). Multiplex CRISPR/Cas9-based genome engineering from a single lentiviral vector. Nucleic Acids Res.

[29] Kaminski R, Bella R, Yin C, Otte J, Ferrante P, Gendelman HE (2016). Excision of HIV-1 DNA by gene editing: a proof-of-concept in vivo study. Gene Ther.

[30] Gao Z, Fan M, Das AT, Herrera-Carrillo E, Berkhout B (2020). Extinction of all infectious HIV in cell culture by the CRISPR-Cas12a system with only a single crRNA. Nucleic Acids Res.

[31] Nguyen H, Wilson H, Jayakumar S, Kulkarni V, Kulkarni S (2021). Efficient inhibition of HIV using CRISPR/Cas13d nuclease system. Viruses.

[32] Qin W, Dion SL, Kutny PM, Zhang Y, Cheng AW, Jillette NL, Malhotra A, Geurts AM, Chen YG, Wang H (2015). Efficient CRISPR/Cas9-mediated genome editing in mice by zygote electroporation of nuclease. Genetics.

[33] Kim S, Kim D, Cho SW, Kim J, Kim JS (2014). Highly efficient RNA-guided genome editing in human cells via delivery of purified Cas9 ribonucleoproteins. Genome Res.

[34] Yang H, Wang H, Shivalila CS, Cheng AW, Shi L, Jaenisch R (2013). One-step generation of mice carrying reporter and conditional alleles by CRISPR/Cas-mediated genome engineering. Cell.

[35] Sun W, Ji W, Hall JM, Hu Q, Wang C, Beisel CL, Gu Z (2015). Self-assembled DNA nanoclews for the efficient delivery of CRISPR-Cas9 for genome editing. Angew Chem Int Ed Engl.

[36] Raveux A, Vandormael-Pournin S, Cohen-Tannoudji M (2017). Optimization of the production of knock-in alleles by CRISPR/Cas9 microinjection into the mouse zygote. Sci Rep.

[37] Chuang CK, Chen CH, Huang CL, Su YH, Peng SH, Lin TY, Tai HC, Yang TS, Tu CF (2017). Generation of GGTA1 mutant pigs by direct pronuclear microinjection of CRISPR/Cas9 plasmid vectors. Anim Biotechnol.

[38] Li L, He ZY, Wei XW, Gao GP, Wei YQ (2015). Challenges in CRISPR/CAS9 delivery: potential roles of nonviral vectors. Hum Gene Ther.

[39] Gori JL, Hsu PD, Maeder ML, Shen S, Welstead GG, Bumcrot D (2015). Delivery and specificity of CRISPR-Cas9 genome editing technologies for human gene therapy. Hum Gene Ther.

[40] Kumar M, Keller B, Makalou N, Sutton RE (2001). Systematic determination of the packaging limit of lentiviral vectors. Hum Gene Ther.

[41] Al Yacoub N, Romanowska M, Haritonova N, Foerster J (2007). Optimized production and concentration of lentiviral vectors containing large inserts. J Gene Med..

[42] Senís E, Fatouros C, Große S, Wiedtke E, Niopek D, Mueller AK, Börner K, Grimm D (2014). CRISPR/Cas9-mediated genome engineering: an adeno-associated viral (AAV) vector toolbox. Biotechnol J.

[43] Ran FA, Cong L, Yan WX, Scott DA, Gootenberg JS, Kriz AJ (2015). In vivo genome editing using *Staphylococcus*
*aureus* Cas9. Nature.

[44] Herskovitz J, Hasan M, Patel M, Blomberg WR, Cohen JD, Machhi J (2021). CRISPR-Cas9 mediated exonic disruption for HIV-1 elimination. EBioMedicine.

[45] Petris G, Casini A, Montagna C (2017). Hit and go CAS9 delivered through a lentiviral based self-limiting circuit. Nat Commun.

[46] Liu Y, Jeeninga RE, Klaver B, Berkhout B, Das AT (2021). Transient CRISPR-Cas treatment can prevent reactivation of HIV-1 replication in a latently infected T-cell line. Viruses.

[47] Liao HK, Gu Y, Diaz A (2015). Use of the CRISPR/Cas9 system as an intracellular defense against HIV-1 infection in human cells. Nat Commun.

[48] Kaminski R, Chen Y, Salkind J (2016). Negative feedback regulation of HIV-1 by gene editing strategy. Sci Rep.

[49] Hu W, Kaminski R, Yang F, Zhang Y, Cosentino L, Li F (2014). RNA-directed gene editing specifically eradicates latent and prevents new HIV-1 infection. Proc Natl Acad Sci U S A.

[50] Zhu W, Lei R, Le Duff Y, Li J, Guo F, Wainberg MA, Liang C (2015). The CRISPR/Cas9 system inactivates latent HIV-1 proviral DNA. Retrovirology.

[51] Chung CH, Allen AG, Atkins AJ, Sullivan NT, Homan G, Costello R (2020). Safe CRISPR-Cas9 inhibition of HIV-1 with high specificity and broad-spectrum activity by targeting LTR NF-κB binding sites. Mol Ther Nucleic Acids.

[52] Lebbink RJ, de Jong DC, Wolters F, Kruse EM, van Ham PM, Wiertz EJ, Nijhuis M (2017). A combinational CRISPR/Cas9 gene-editing approach can halt HIV replication and prevent viral escape. Sci Rep.

[53] Binda CS, Klaver B, Berkhout B, Das AT (2020). CRISPR-Cas9 dual-gRNA attack causes mutation, excision and inversion of the HIV-1 proviral DNA. Viruses.

[54] Ophinni Y, Miki S, Hayashi Y, Kameoka M (2020). Multiplexed tat-targeting CRISPR-Cas9 protects T cells from acute HIV-1 infection with inhibition of viral escape. Viruses.

[55] Yin L, Hu S, Mei S, Sun H, Xu F, Li J (2018). CRISPR/Cas9 inhibits multiple steps of HIV-1 infection. Hum Gene Ther.

[56] Wang Q, Liu S, Liu Z, Ke Z, Li C, Yu X (2018). Genome scale screening identification of SaCas9/gRNAs for targeting HIV-1 provirus and suppression of HIV-1 infection. Virus Res.

[57] Magro G, Calistri A, Parolin C (2021). Targeting and understanding HIV latency: the CRISPR system against the provirus. Pathogens.

[58] Yin L, Zhao F, Sun H, Wang Z, Huang Y, Zhu W (2020). CRISPR-Cas13a inhibits HIV-1 Infection. Mol Ther Nucleic Acids.

[59] Yin C, Zhang T, Qu X, Zhang Y, Putatunda R, Xiao X (2017). In vivo excision of HIV-1 provirus by saCas9 and multiplex single-guide RNAs in animal models. Mol Ther.

[60] Bella R, Kaminski R, Mancuso P, Young WB, Chen C, Sariyer R (2018). Removal of HIV DNA by CRISPR from patient blood engrafts in humanized mice. Mol Ther Nucleic Acids..

[61] Mancuso P, Chen C, Kaminski R, Gordon J, Liao S, Robinson JA (2020). CRISPR based editing of SIV proviral DNA in ART treated non-human primates. Nat Commun.

[62] Dash PK, Kaminski R, Bella R, Su H, Mathews S, Ahooyi TM (2019). Sequential LASER ART and CRISPR treatments eliminate HIV-1 in a subset of infected humanized mice. Nat Commun.

[63] Sengupta S, Siliciano RF (2018). Targeting the latent reservoir for HIV-1. Immunity.

[64] Kim Y, Anderson JL, Lewin SR (2018). Getting the "kill" into "shock and kill": strategies to eliminate latent HIV. Cell Host Microbe.

[65] Bullen CK, Laird GM, Durand CM, Siliciano JD, Siliciano RF (2014). New ex vivo approaches distinguish effective and ineffective single agents for reversing HIV-1 latency in vivo. Nat Med.

[66] Saayman S, Ali SA, Morris KV, Weinberg MS (2015). The therapeutic application of CRISPR/Cas9 technologies for HIV. Expert Opin Biol Ther.

[67] Zhang Y, Yin C, Zhang T, Li F, Yang W, Kaminski R (2015). CRISPR/gRNA-directed synergistic activation mediator (SAM) induces specific, persistent and robust reactivation of the HIV-1 latent reservoirs. Sci Rep.

[68] Bialek JK, Dunay GA, Voges M, Schäfer C, Spohn M, Stucka R (2016). Targeted HIV-1 Latency Reversal Using CRISPR/Cas9-Derived Transcriptional Activator Systems. PLoS ONE.

[69] Limsirichai P, Gaj T, Schaffer DV (2016). CRISPR-mediated activation of latent HIV-1 expression. Mol Ther.

[70] Saayman SM, Lazar DC, Scott TA, Hart JR, Takahashi M, Burnett JC (2016). Potent and targeted activation of latent HIV-1 using the CRISPR/dCas9 activator complex. Mol Ther.

[71] Vansant G, Bruggemans A, Janssens J, Debyser Z (2020). Block-and-lock strategies to cure HIV infection. Viruses.

[72] Olson A, Basukala B, Lee S, Gagne M, Wong WW, Henderson AJ (2020). Targeted chromatinization and repression of HIV-1 provirus transcription with repurposed CRISPR/Cas9. Viruses.

[73] Doench JG, Fusi N, Sullender M, Hegde M, Vaimberg EW, Donovan KF (2016). Optimized sgRNA design to maximize activity and minimize off-target effects of CRISPR-Cas9. Nat Biotechnol.

[74] Das AT, Binda CS, Berkhout B (2019). Elimination of infectious HIV DNA by CRISPR-Cas9. Curr Opin Virol.

[75] Wang G, Zhao N, Berkhout B, Das AT (2016). CRISPR-Cas9 can inhibit HIV-1 replication but NHEJ repair facilitates virus escape. Mol Ther.

[76] Wang Z, Pan Q, Gendron P, Zhu W, Guo F, Cen S (2016). CRISPR/Cas9-derived mutations both inhibit HIV-1 replication and accelerate viral escape. Cell Rep.

[77] Liang C, Wainberg MA, Das AT, Berkhout B (2016). CRISPR/Cas9: a double-edged sword when used to combat HIV infection. Retrovirology.

[78] Herrera-Carrillo E, Gao Z, Berkhout B (2020). CRISPR therapy towards an HIV cure. Brief Funct Genomics.

[79] Kimberland ML, Hou W, Alfonso-Pecchio A, Wilson S, Rao Y, Zhang S, Lu Q (2018). Strategies for controlling CRISPR/Cas9 off-target effects and biological variations in mammalian genome editing experiments. J Biotechnol.

[80] Duan J, Lu G, Xie Z, Lou M, Luo J, Guo L, Zhang Y (2014). Genome-wide identification of CRISPR/Cas9 off-targets in human genome. Cell Res.

[81] Wang X, Wang Y, Wu X, Wang J, Wang Y, Qiu Z (2015). Unbiased detection of off-target cleavage by CRISPR-Cas9 and TALENs using integrase-defective lentiviral vectors. Nat Biotechnol.

[82] Fu Y, Sander JD, Reyon D, Cascio VM, Joung JK (2014). Improving CRISPR-Cas nuclease specificity using truncated guide RNAs. Nat Biotechnol.

[83] Ran FA, Hsu PD, Lin CY, Gootenberg JS, Konermann S, Trevino AE (2013). Double nicking by RNA-guided CRISPR Cas9 for enhanced genome editing specificity. Cell.

[84] Tsai SQ, Wyvekens N, Khayter C, Foden JA, Thapar V, Reyon D (2014). Dimeric CRISPR RNA-guided FokI nucleases for highly specific genome editing. Nat Biotechnol.

